# P-2112. Development and Validation of a Cheap, Portable Assay to Rapidly Profile Bacterial Infections of the Bloodstream

**DOI:** 10.1093/ofid/ofae631.2268

**Published:** 2025-01-29

**Authors:** David Roach, Roby P Bhattacharyya, Pun Sangruji, Shriya Bhat, Selama Tesfamariam

**Affiliations:** Brigham and Women's Hospital, Broad Institute of MIT and Harvard, Boston, Massachusetts; Massachusetts General Hospital, Cambridge, Massachusetts; Broad Institute, Tufts University, Cambridge, Massachusetts; Broad Institute, Harvard College, Cambridge, Massachusetts; Broad Institute, Howard University, Washington, DC, District of Columbia

## Abstract

**Background:**

Antimicrobial resistant (AMR) bacterial infections pose a significant and growing health threat worldwide, and their burden is most severe in lower income areas where diagnostic infrastructure is lacking. The development of cost-effective and accessible diagnostic tools for the rapid identification of resistant infections in low-resource settings is crucial. Specific High-sensitivity Enzymatic Reporter Unlocking, or SHERLOCK, is a promising CRISPR-based system that can aid in the diagnosis of infections and support clinical decision making. Here, we present an assay termed BADLOCK (Bacteria and AMR Detection by SHERLOCK) that uses this technology to rapidly identify bacterial species and AMR genes on blood cultures.

BADLOCK workflow demonstrating simplicity of approach and rapid turnaround.

**Figure:**
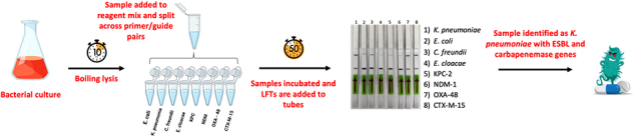

**Methods:**

We combine isothermal amplification of targets with CRISPR/Cas13-based detection into a single step, allowing for a streamlined workflow and a total time-to-identification of less than two hours from positive cultures using lateral flow strips (Fig. 1). The assay primarily targets *topA* for species identification, a highly conserved gene within species with sufficient variability between them to enable species-level typing. A panel of clinically important AMR genes, including the ESBL *bla_CTX-M-15_*, three major carbapenemases, and the GPC resistance genes *mecA* and *vanA*, is also included.

Pilot study assessing assay performance.

**Figure:**
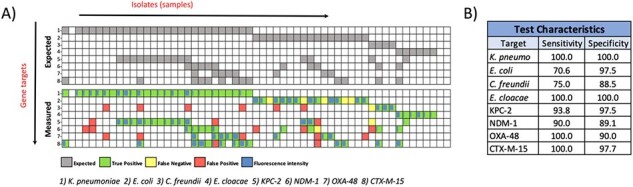

A) each column represents a different isolate while each row represents a different CRISPR gene target, which are enumerated at the bottom. Each species name refers to the topA gene sequence unique to that species. The top box shows the expected gene content based on prior sequencing, while the bottom box shows the results of each assay. The blue shading shows relative fluorescence intensity of the generated signal. B) Basic test characteristics for each target gene.

**Results:**

In addition to the AMR resistance genes, we have validated a panel of 9 gram-negative rods, 6 staphylococci, 2 enterococci, and 2 streptococcal species with further targets being developed, and have performed a laboratory pilot study on a subset of these (Fig. 2). We have additionally collected over 600 sequential positive blood cultures and are in the process of performing a large clinical validation study on patient samples, thereby elucidating the precise test characteristics of our assay.

**Conclusion:**

We have developed an assay capable of rapidly detecting bacterial species causing bloodstream infections and their major epidemic AMR genes on low-cost paper strips. Our work has the potential to improve the diagnostic landscape for resistant infections in low-and-middle-income countries and serve as an adjunctive diagnostic to traditional approaches in highly resourced setting, which would represent a major step forward in infectious disease diagnostics.

**Disclosures:**

All Authors: No reported disclosures

